# The Test Your Memory for Mild Cognitive Impairment (TYM-MCI)

**DOI:** 10.1136/jnnp-2016-315327

**Published:** 2017-09-14

**Authors:** Jeremy M Brown, Claire J Lansdall, Julie Wiggins, Kate E Dawson, Kristina Hunter, James B Rowe, Richard A Parker

**Affiliations:** 1 Department of Neurology, Cambridge University Hospitals, Hills Road, Cambridge, UK; 2 Queen Elizabeth Hospital NHS Trust, Gayton Road, King’s Lynn, Norfolk, UK; 3 Cambridge University Department of Clinical Neurosciences, Cambridge, UK; 4 Edinburgh Clinical Trials Unit, Usher Institute of Population Health Sciences and Informatics, University of Edinburgh, Edinburgh, UK

**Keywords:** TYM-MCI, TYM, amnestic mild cognitive impairment, cognition

## Abstract

**Background:**

To validate a short cognitive test: the Test Your Memory for Mild Cognitive Impairment (TYM-MCI) in the diagnosis of patients with amnestic mild cognitive impairment or mild Alzheimer’s disease (aMCI/AD).

**Methods:**

Two hundred and two patients with mild memory problems were recruited. All had ‘passed’ the Mini-Mental State Examination (MMSE). Patients completed the TYM-MCI, the Test Your Memory test (TYM), MMSE and revised Addenbrooke’s Cognitive Examination (ACE-R), had a neurological examination, clinical diagnostics and multidisciplinary team review.

**Results:**

As a single test, the TYM-MCI performed as well as the ACE-R in the distinction of patients with aMCI/AD from patients with subjective memory impairment with a sensitivity of 0.79 and specificity of 0.91. Used in combination with the ACE-R, it provided additional value and identified almost all cases of aMCI/AD. The TYM-MCI correctly classified most patients who had equivocal ACE-R scores. Integrated discriminant improvement analysis showed that the TYM-MCI added value to the conventional memory assessment. Patients initially diagnosed as unknown or with subjective memory impairment who were later rediagnosed with aMCI/AD scored poorly on their original TYM-MCI.

**Conclusion:**

The TYM-MCI is a powerful short cognitive test that examines verbal and visual recall and is a valuable addition to the assessment of patients with aMCI/AD. It is simple and cheap to administer and requires minimal staff time and training.

## Introduction

Early accurate diagnosis of Alzheimer’s disease (AD) is a major global health priority.^1^ Early diagnosis is important for patients and helps plan care. Emerging disease-modifying treatments for AD are likely to halt disease progression rather than reverse changes, so it is becoming critical to identify AD early. Patients with AD typically present with symptoms caused by poor recall of recently learnt visual and verbal material; clinical testing reveals episodic memory deficits.^2 3^ Patients presenting with these symptoms and signs are diagnosed as having amnestic mild cognitive impairment (aMCI) if their problems are mild (with intact functional abilities)^4^ or probable AD if they have problems which impact on their lives.^5^ Many patients with aMCI progress to AD^6^ and patients with a diagnosis of aMCI or mild AD form a continuum, so we consider them as one group: aMCI/AD.

The diagnosis of cognitive disease requires a history from the patient and an informant, an examination including short cognitive tests and investigation.^7^ Short cognitive tests can be divided into three groups: orientation-dominated questionnaires, highly selective tests and multidomain tests.^8^ Multidomain tests are the most useful in aiding diagnosis.^8^ The most widely used short cognitive tests in memory clinics include the Addenbrooke’s Cognitive Examinations (ACE-R).^9 10^

A clinical assessment including a multidomain cognitive test usually enables diagnosis in patients with moderate AD. In milder cases, the diagnosis of AD is more difficult and may remain unclear after initial assessment. There are several methods available to clarify the diagnosis in mild cases including expert neuropsychological assessment, cerebrospinal fluid biomarkers, structural and functional brain imaging (including amyloid positron emission tomography). These are useful but share disadvantages: they require referral to specialists, take time and are expensive. This limits them to specialist services within highly developed countries. This study examines whether a targeted short cognitive test examining visual and verbal recall could be a cheap, effective method of supporting a diagnosis of aMCI/AD.

Some current multidomain short cognitive tests such as the ACEs,^9 10^ Montreal Cognitive Assessment (MOCA)^11^ and the Test Your Memory (TYM)^12 13^ are useful in patients with aMCI, but these tests are designed for several purposes and do not test visual recall (a cognitive skill affected early in AD). The Test Your Memory for Mild Cognitive Impairment (TYM-MCI), formerly named the H-TYM, was designed to test verbal and visual recall to optimise its sensitivity to aMCI/AD. The TYM-MCI is shown in online supplementary appendix 1.

10.1136/jnnp-2016-315327.supp1 Supplementary Appendix 1

In the validation study, the TYM-MCI was shown to be very powerful in distinguishing patients with mild AD from normal controls with excellent sensitivity (0.95) and specificity (0.93).^14^ This study examines the performance of the TYM-MCI in the more demanding and clinically relevant task of distinguishing patients with aMCI/AD from patients with subjective memory complaints (SMC) without neurological disease (for example, patients diagnosed as worried well or depressed).

## Materials and methods

### Setting

Patients were enrolled from a UK memory clinic between March 2011 and March 2014. A prospective cohort design was used to compare the TYM-MCI with the clinical diagnosis in patients supported as indicated by imaging, neuropsychological assessment and follow-up. Patients were recruited prospectively on a convenience basis—when nurses had time. The 96 patients with SMC and 58 patients with aMCI/AD recruited in this study provide 84% power to detect a difference of 2 points in the TYM-MCI score assuming a two-sided 5% significance level and a SD of 4 based on a previous study.^12^

Patients were seen by a consultant neurologist and/or psychiatrist. All had a physical examination and completed the ACE-R, Mini-Mental State Examinations (MMSE),^15^ TYM test and TYM-MCI. The diagnoses of AD and aMCI were made according to current diagnostic criteria^4 5^ and were agreed at a multidisciplinary team (MDT) meeting; other diagnoses were made by consensus at the MDT. The specialist and the MDT had access to the ACE-R score but not the TYM or TYM-MCI. Follow-up and investigations were determined on clinical need at the MDT meeting; 57% of the patients were followed up for between 4 months and 3 years. To select patients with mild problems, those scoring ≤24/30 on the MMSE were excluded.

Ethical permission for the study was obtained from Cambridgeshire 2 Ethics Committee.

Patients were divided into six diagnostic groups:

1. SMC
2. aMCI/AD
3. No clear diagnosis
4. Fixed memory deficit head injury
5. Neurological disease sometimes associated with cognitive problems, for example, epilepsy
6. Other degenerative dementia

The analysis focuses on groups 1 and 2. Patients with mild non-amnestic cognitive problems were included in groups 3 to 5 rather than treated as a separate group.

### Administering and scoring the TYM-MCI

The TYM-MCI was assessed in five ways:

1. As a single test in the diagnosis of aMCI/AD.
2. As a combined assessment with the ACE-R.
3. In patients with equivocal ACE-R scores.
4. Whether it adds value to the normal memory clinic assessment.
5. As a predictor of patients initially reassured but who are later rediagnosed with aMCI/AD.

The ordinary TYM test was also administered. To avoid confusion between the tests, the performance of the TYM test is in online supplementary appendix 2.

10.1136/jnnp-2016-315327.supp2 Supplementary Appendix 2

The TYM-MCI was printed on both sides of a sheet of card (see online supplementary appendix 1).

The TYM-MCI was supervised by a nurse in a quiet room. Training the nurses took 5 min. The patient was asked to copy the diagram and read the passage silently and then aloud. The patient answered the page 1 questions referring back to the passage. The patient was not told to memorise the answers. The card was turned over and the patient was asked to:

- reproduce the diagram within the square
- answer the same questions
- circle flowers mentioned in the passage
- answer new questions concerning the passage.

### Scoring

Five page 2 recall tasks were scored:

1. Recall of the drawing           /15
2. Recall of page one answers          /5
3. Recognition of flowers from the passage   /4
4. No flower misrecognition          /2
5. Answering new questions on the passage    /4
6. Total                    /30

The page two score=TYM-MCI score. Verbal recall 1 is the recall of answers already given, and verbal recall 2 is the new answers.

The cut-off of ≤13/30 optimised in the validation study^14^ was used. The TYM-MCI, TYM and ACE-R were scored by the nurses with access to some clinical information.

### Statistical methods

Cronbach’s alpha was calculated for the TYM-MCI.

The distribution of the test scores did not follow a normal distribution so the Mann-Whitney U test was applied to compare between groups. Data from patients with SMC and aMCI/AD were used to plot a receiver operating characteristic (ROC) curve. Sensitivities, specificities and predictive values were calculated using standard methods. An ‘or’ rule was used for combined ROC analysis of the ACE-R and TYM-MCI. Correlations between the TYM-MCI and ACE-R were calculated using Spearman’s r coefficient for non-parametric data.

To produce the age-matched groups, an age range of 51–80 years was set. The median value was obtained for each group, and all cases above the median were included for the SMC group and all cases below the median were included for the aMCI/AD group. Cases were then randomly sampled from the remaining cases to form equal sample sizes of 50.

To determine whether the TYM-MCI provided additional information to standard tests, logistic regression was used to assess predictive ability. A logistic regression model was fitted to outcome (aMCI/AD or SMC) adjusting for age, MMSE and ACE-R. The integrated discriminant improvement^16^ was calculated to assess the improvement in model performance when the TYM-MCI is included compared with when it is excluded. Additional ROC curves were plotted using the predictive values generated from the models.

## Results

### Patient characteristics

Two hundred and two patients aged between 40 years and 80 years were enrolled. Two patients (one with SMC, one with aMCI) failed to complete the tests and were excluded. The characteristics of the remaining 200 are shown (table 1). There were no adverse events. Patients gave informed consent. The patients were divided into the six groups described, but principal analyses were restricted to the SMC and aMCI/AD groups in keeping with the primary aim of the study. The SMC patients formed the largest group with a mean age of 59.2 years, significantly lower than aMCI/AD patients (mean age 67.8 years). Therefore, additional analyses were performed on age-matched subsamples from the SMC and aMCI/groups.

**Table 1:** Characteristics and performance of the six groups on short cognitive tests including mean scores and SD

Group	Diagnosis	Patients (n)	Mean age	MMSE/30	ACE-R/100	TYM-MCI/30
1	SMC	96	59.2 (SD 9.2)	28.6 (SD 1.1)	90.8 (SD 5.5)	19.8 (SD 5.0)
2	aMCI/AD	58	67.8 (SD 7.4)	26.8 (SD 1.5)	81.3 (SD 5.3)	9.4 (SD 4.6)
3	No clear diagnosis	13	66.5 (SD 8.6)	27.1 (SD 1.7)	82.0 (SD 6.4)	14.9 (SD 4.6)
4	Fixed deficits	11	61.7 (SD 8.8)	27.6 (SD 1.5)	84.0 (SD 6.0)	13.4 (SD 7.4)
5	Other neurological disease	12	61.9 (SD 9.8)	28.5 (SD 1.4)	88.2 (SD 6.0)	16.8 (SD 5.6)
6	Non-AD dementia	10	66.6 (SD 12.5)	27.4 (SD 1.7)	82.8 (SD 7.1)	14.0 (SD 4.3)

ACE-R, Addenbrooke’s Cognitive Examinations; aMCI/AD, amnestic mild cognitive impairment or Alzheimer’s disease; MMSE, Mini-Mental State Examinations; SMC, subjective memory complaints; TYM-MCI, Test Your Memory for Mild Cognitive Impairment.

### Reliability of the TYM-MCI

Cronbach’s alpha was 0.89 showing high internal consistency of the TYM-MCI.

#### The TYM-MCI and ACE-R as single tests to distinguish patients who have aMCI/AD from patients with SMC

Both the TYM-MCI and ACE-R detected a significant difference between patients with aMCI/AD and patients with SMC; the differences were proportionally larger in the TYM-MCI than the ACE-R (table 2). Effect size shows a proportionally larger difference for the visual recall task and the overall TYM-MCI score compared with the ACE-R. Spearman’s rho coefficient for non-parametric data was 0.66 (p<0.001) showing a moderately high correlation between the TYM-MCI and ACE-R.

**Table 2:** Comparison of the performance of the ACE-R, TYM-MCI subtests and TYM-MCI scores using effect size from a two-sample t-test and Mann-Whitney U test p value in patients with aMCI/AD and those with SMC

	Subjective memory complaints (mean (SD))	Amnestic MCI/mild AD (mean (SD))	Effect size*	p Value Mann-Whitney U test
ACE-R	90.8 (5.5)	81.3 (5.3)	11.0	<0.001
Overall TYM-MCI	19.8 (5.0)	9.4 (4.6)	13.0	<0.001
Visual recall	9.8 (3.3)	2.9 (3.1)	13.0	<0.001
Verbal recall 1	4.4 (0.9)	3.1 (1.1)	8.5	<0.001
Verbal recall 2	5.5 (2.4)	3.4 (2.2)	5.4	<0.001

*Mean difference divided by SE (effect size from a two sample t-test).

ACE-R, Addenbrooke’s Cognitive Examinations; AD, Alzheimer’s disease, aMCI/AD, amnestic mild cognitive impairment or Alzheimer’s disease; TYM-MCI, Test Your Memory for Mild Cognitive Impairment.

**Figure 1:**
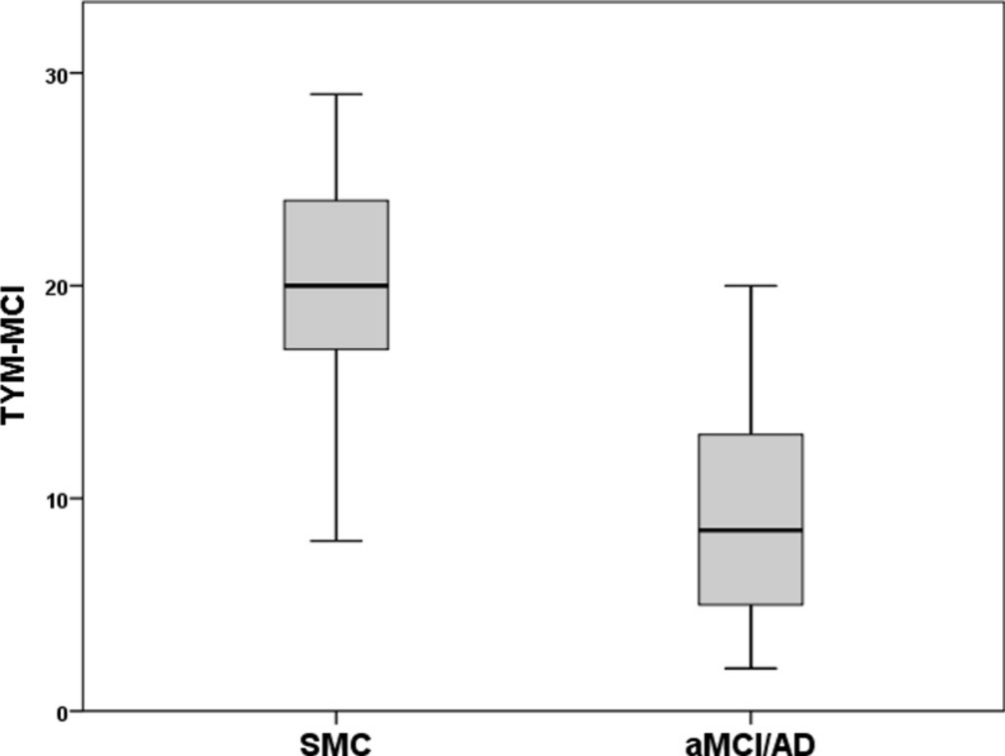
Box plot showing the distribution of the TYM-MCI score for patients with SMC and aMCI/AD. aMCI/AD, amnestic mild cognitive impairment or Alzheimer’s disease; SMC, subjective memory complaints; TYM-MCI, Test Your Memory for Mild Cognitive Impairment.

**Figure 2:**
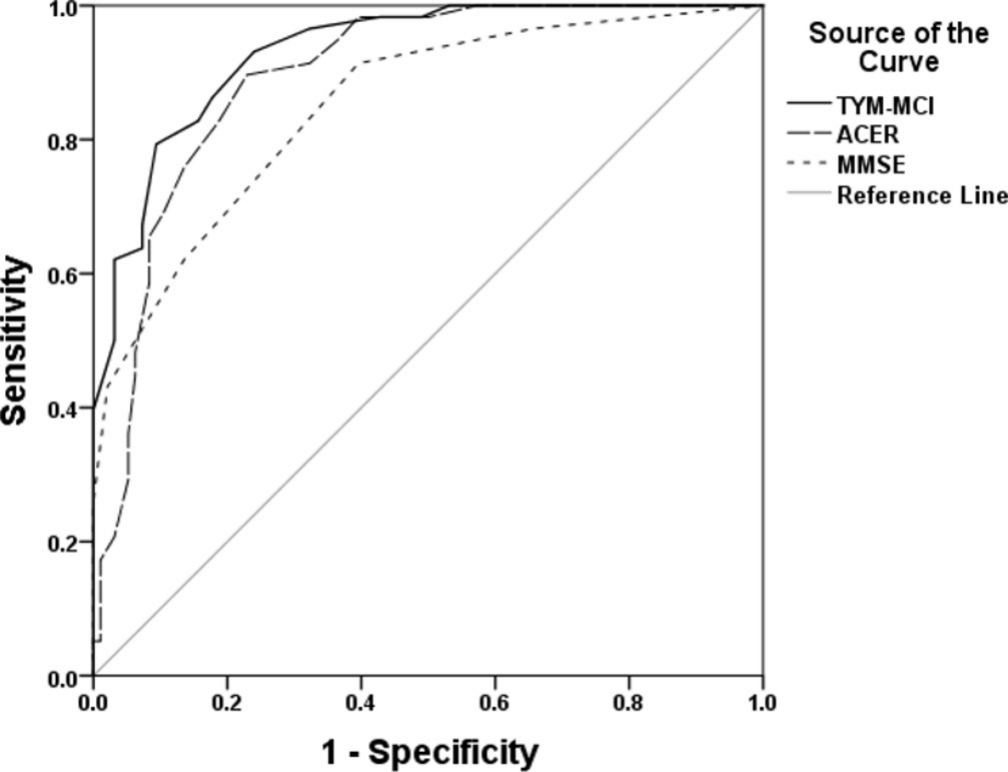
ROC curve for the TYM-MCI, ACE-R and MMSE in the separation of patients with aMCI/AD from those with SMC. ACE-R, Addenbrooke’s Cognitive Examinations; aMCI/AD, amnestic mild cognitive impairment or Alzheimer’s disease; MMSE, Mini-Mental State Examinations; TYM-MCI, Test Your Memory for Mild Cognitive Impairment.

The difference between patients with SMC and patients with aMCI/AD on the TYM-MCI is shown in figure 1. There is a clear separation of most individuals: only patients with SMC scored ≥20 and only patients with aMCI/AD scored ≤8. The ROC curve (figure 2) compares the ability of the TYM-MCI, ACE-R and MMSE to distinguish patients with aMCI/AD from those with SMC. Sensitivities, specificities, positive predicative value (PPV) and negative predicative value (NPV) for the TYM-MCI and ACE-R are shown in table 3.

**Table 3:** Sensitivity and specificity of the ACE-R and TYM-MCI

	Specificity	Sensitivity	False negatives (n)	False positives (n)	PPV	NPV	AUC of continuous score
ACE-R≤82	0.92 (0.84–0.96)	0.59 (0.45–0.71)	24	8	0.81 (0.66–0.91)	0.79 (0.70–0.86)	0.89 (0.84–0.94)
ACE-R≤87	0.77 (0.67–0.85)	0.90 (0.79–0.96)	6	22	0.70 (0.59–0.80)	0.92 (0.84–0.97)	0.89 (0.84–0.94)
TYM-MCI≤13	0.91 (0.83–0.96)	0.79 (0.67–0.89)	12	9	0.84 (0.71–0.92)	0.88 (0.80–0.94)	0.93 (0.89–0.97)

95% confidence limits in brackets.

ACE-R, Addenbrooke’s Cognitive Examinations; AUC, area under the curve; NPV, negative predicative value; PPV, positive predicative value; TYM-MCI, Test Your Memory for Mild Cognitive Impairment.

All tasks showed a significant difference between SMC patients and aMCI/AD patients (table 2). The largest difference was for visual recall as in the original study.^14^

##### Analysis of age-matched groups

As there was a significant difference in age between the SMC and aMCI/AD groups, further analyses were performed using age-matched cohorts (tables 4 and 5). Non-parametric Mann-Whitney U test showed no significant difference in the ages of the revised groups (SMC average age 64.4 years, aMCI/AD 66.7 years p=0.23).

**Table 4:** Comparison of age-matched groups

Short Cognitive Test or subtest	Patients with subjective memory complaints (mean (SD))	Patients with aMCI/AD (mean (SD))	Effect size*	p Score Mann-Whitney U test
ACE-R	91.1 (5.4)	81.4 (5.5)	9.4	p<0.001
Total TYM-MCI	19.3 (5.2)	9.5 (4.7)	10.3	p<0.001
Visual recall	9.4 (3.3)	3.1 (3.2)	10.3	p<0.001
Verbal recall 1	4.3 (1.2)	3.1 (1.1)	6.0	p<0.001
Verbal recall 2	5.5 (2.6)	3.4 (2.2)	4.6	p<0.001

*Mean difference divided by standard error (effect size from a two sample t-test).

ACE-R, Addenbrooke’s Cognitive Examinations; aMCI/AD, amnestic mild cognitive impairment or Alzheimer’s disease; TYM-MCI, Test Your Memory for Mild Cognitive Impairment.

The area under the curve (AUC) of the ROC curve was 0.91 (95% confidence limit (Cl) 0.86 to0.96) for the TYM-MCI, compared with 0.89 (95% Cl 0.78 to 0.96) for the ACE-R. Using the cut-off of ≤13, the sensitivity was 0.78 (95% Cl 0.65 to 0.88) and specificity 0.89 (95% Cl 0.78 to 0.96). This resulted in a PPV of 0.88 (95% Cl 0.75 to 0.95) and a NPV of 0.80 (95% Cl 0.68 to 0.89) for the TYM-MCI.

**Table 5:** Sensitivity and specificity of the ACE-R and H-TYM in age-matched patients. 95% confidence limits in brackets

	Specificity	Sensitivity	False negatives (n)	False positives (n)	PPV	NPV	AUC of continuous score
ACE-R≤82	0.91 (0.80 to 0.97)	0.56 (0.42 to 0.70)	24	5	0.86 (0.71 to 0.95)	0.68 (0.56 to 0.78)	0.90 (0.84 to 0.96)
ACE-R≤87	0.82 (0.69 to 0.91)	0.89 (0.78 to 0.96)	6	10	0.83 (0.71 to 0.92)	0.88 (0.76 to 0.96)	0.90 (0.84 to 0.96)
TYM-MCI≤13	0.89 (0.78 to 0.96)	0.78 (0.65 to 0.88)	12	6	0.88 (0.75 to 0.95)	0.80 (0.68 to 0.89)	0.91 (0.86 to 0.96)

ACE-R, Addenbrooke’s Cognitive Examinations; NPV, negative predicative value; PPV, positive predicative value; TYM-MCI, Test Your Memory for Mild Cognitive Impairment.

#### Analysis of the TYM-MCI and ACE-R in combination

The cross-classification of TYM-MCI and ACE-R scores against the clinical diagnosis is shown (table 6). Combining the results of the TYM-MCI with the ACE-R with an ‘or’ rule, the tests show a superior performance to either test alone. Using the criteria of scoring ≤82 on the ACE-R or scoring ≤13 on the TYM-MCI then the results are excellent with a sensitivity of 88% (95% Cl 77% to 95%) and specificity of 85% (95% Cl 77% to 92%). Where it is crucial that no cases of aMCI/AD are missed then combining the ACE-R (cut-off 87) with the TYM-MCI yields a sensitivity of 97% (95% Cl 88% to 100%).

**Table 6:** Mean scores of the ACE-R cross-classified against the TYM-MCI and the final diagnosis of subjective memory complaint

		TYM-MCI score		
		0–13	14+	Total
(A) Patient with SMC				
ACE-R score	0–82	3	5	8
	83–87	3	11	14
	88+	3	71	74
	Total	9	87	96
(B) Patient with aMCI/AD				
ACE-R score	0–82	29	5	34
	83–87	13	5	18
	88+	4	2	6
	Total	46	12	58

ACE-R, Addenbrooke’s Cognitive Examinations; aMCI/AD, amnestic mild cognitive impairment or Alzheimer’s disease; SMC, subjective memory complaint; TYM-MCI, Test Your Memory for Mild Cognitive Impairment.

#### Analysis of TYM-MCI in patients with equivocal ACE-R scores

The analysis on the 32 patients with equivocal ACE-R scores (83–87) showed that the TYM-MCI was very useful in this group. Mean score on the TYM-MCI was 17.2 for patients with SMC, 9.9 for patients with aMCI/AD. Eleven of fourteen patients with SMC and equivocal ACE-R scores passed the TYM-MCI. Thirteen of eighteen patients with aMCI/AD with equivocal ACE-R scores were detected by the TYM-MCI. The visual recall task was particularly useful (mean score 8.2 in patients with SMC, 3.2 in patients with aMCI/AD). The AUC of the ROC was 0.85 with a sensitivity of 0.72 and specificity of 0.79 (cut-off ≤13). Using a cut-off of ≤18, all cases of aMCI/AD with equivocal ACE-R scores were detected by the TYM-MCI.

#### The TYM-MCI as an additional test to the ACE-R

Logistic regression analysis with integrated discriminant improvement analysis (table 7) showed that the TYM-MCI score is a highly significant predictor of the diagnosis of aMCI/AD (p<0.0001) even after adjusting for the ACE-R score, MMSE and age. With model calibration, the logistic regression model which includes the TYM-MCI score confers an advantage over the model excluding the TYM-MCI: Nagelkerke’s R^2^ statistic increased from 0.70 to 0.77 after including the TYM-MCI score and the Hosmer-Lemeshow χ^2^ statistic decreased from 8.02 (p=0.43) to 5.52 (p=0.70). ROC curves were plotted based on the model predictive values for each of the models. The AUC was 0.96 (Cl 0.94 to 0.99) for the model including the TYM-MCI score, compared with 0.93 (95% Cl 0.90 to 0.97) for the model without the TYM-MCI.

**Table 7:** Logistic regression analysis to assess the predictive ability  of the TYM-MCI, MMSE, ACE-R and age

	OR	95% CI	p Value
TYM-MCI score	0.75	0.65 to 0.86	p<0.0001
MMSE	0.82	0.47 to 0.97	p=0.51
ACE-R	0.85	0.75 to 0.97	p=0.017
Age	1.12	1.04 to 1.20	p=0.002

ACE-R, Addenbrooke’s Cognitive Examinations; MMSE, Mini-Mental State Examinations; TYM-MCI, Test Your Memory for Mild Cognitive Impairment.

Integrated discriminant improvement (IDI) was used^16^ to quantify the significance of this difference using the new model including the TYM-MCI compared with the model without the TYM-MCI. The IDI was 0.10 (95% confidence limits 0.05 to 0.16, p<0.001) showing that the TYM-MCI provides a significant improvement in model performance.

## Analysis of patients in whom the diagnosis was changed to aMCI/AD

Thirteen patients diagnosed as SMC or unknown were subsequently rediagnosed with aMCI/AD (10 were under follow-up, 3 re-referred). These patients had significantly lower scores on their baseline TYM-MCI and ACE-R compared with all patients with SMC (table 8), they performed particularly poorly on visual recall.

**Table 8:** Mean baseline scores for the patients whose diagnosis changed to aMCI/AD compared with patients in group 1 (SMC)

Short Cognitive Test or subtest	Mean scores of patients whose diagnosis changed to aMCI/AD (n=13)	Subjective memory complaints (n=96)	Effect size*	p Score Mann-Whitney U test
ACE-R	83.5 (4.4)	90.8 (5.5)	4.6	p<0.001
Total TYM-MCI	9.8 (4.7)	19.8 (5.0)	6.9	p<0.001
Visual recall	3.4 (2.9)	9.8 (3.3)	6.6	p<0.001
Verbal recall 1	2.7 (1.3)	4.4 (0.9)	6.2	p<0.001
Verbal recall 2	3.7 (2.2)	5.5 (2.4)	2.5	p=0.02

*Mean difference divided by standard error (effect size from a two sample t-test).

ACE-R, Addenbrooke’s Cognitive Examinations; aMCI/AD, amnestic mild cognitive impairment or Alzheimer’s disease; SMC, subjective memory complaint; TYM-MCI, Test Your Memory for Mild Cognitive Impairment.

## Discussion

The TYM-MCI is a targeted short cognitive test designed for a single purpose—the identification of aMCI/AD. The TYM-MCI is free, easy and quick to use and requires minimal training to administer. A previous study showed that the TYM-MCI was very powerful at distinguishing patients with aMCI/AD from healthy controls with an AUC of the ROC of 0.99.^14^ This study showed that the TYM-MCI performed very well on the useful and challenging task of distinguishing patients with aMCI/AD from those with SMC (with diagnoses such as worried well, depression, anxiety and functional problems) with an AUC of 0.93.

A new cohort of patients attending a memory clinic who ‘passed’ the MMSE was recruited. The performance of the TYM-MCI in the diagnosis of aMCI or mild AD was assessed in comparison with and in combination with the ACE-R. The direct comparison of the tests needs to be interpreted with caution as there are two biases: first, the ACE-R and MMSE were available to the clinician whereas the TYM-MCI was not and second only patients who passed the MMSE were included. The first of these confounding factors may favour the ACE-R as the result will influence the specialist. The second precluded a direct comparison with the MMSE noting that it is well established that the MMSE is insensitive to aMCI/AD.^12 17 18^ Patients were recruited in this study when there was sufficient time for nurses to administer tests, which could introduce a selection bias.

Clinicians were often uncertain of the diagnosis initially in this difficult group, but where the balance of evidence from clinical assessment, investigations and follow-up favoured aMCI/AD or SMC, this was recorded. The mean MMSE score of 26.8 in the aMCI/AD group was high, confirming that patients had mild disease. The TYM-MCI showed a complete separation of aMCI/AD and SMC for patients with scores below 8 and above 20. The ACE-R also performed well in this study. There were larger differences between the groups on the TYM-MCI compared with the ACE-R: the TYM-MCI scores of aMCI/AD patients were 53% of the TYM-MCI scores of SMC patients compared with a 10% difference in the ACE-R score. The largest difference was in visual recall. Calculation of the effect size showed better performance of the TYM-MCI and the visual recall subtest compared with the ACE-R in distinguishing aMCI/AD from SMC.

Neuropsychological profiles of AD show that the characteristic earliest features of mild AD are poor recall of recently learnt visual and verbal material and these can be present and appear stable for years in the ‘pre-dementia’ stage of AD pathology.^19^ Although two short tests that are used occasionally test visual recall, the Mini-Mental Parkinson’s^20^ and the visual association test,^21^ visual recall is often ignored in the clinical assessment of suspected AD. The TYM-MCI is the first short cognitive test to test both visual and verbal recall making it a suitable instrument to detect disease in this important group of patients.

Additional analyses performed with age-matched sub-groups from patients with aMCI/AD and SMC showed that the differences remained highly significant for all the TYM-MCI subtests with similar effect sizes, PPV and ROC curve. Logistic regression analysis confirmed that the TYM-MCI result remained significant after adjusting for age.

Addition of the TYM-MCI result to the ACE-R (cut-off 87) improves its performance: only 2 of the 58 aMCI/AD patients passed both tests and only 6 of the 96 SMC patients failed both tests.

Examining the usefulness of the TYM-MCI in the 32 patients with equivocal ACE-R scores showed it performed very well with a high AUC, sensitivity and specificity in distinguishing such patients with aMCI/AD from those with SMC. Logistic regression analysis showed that the TYM-MCI score was a highly significant predictor of aMCI/AD (p<0.0001) even after adjusting for ACE-R, MMSE scores and age. The integrated discriminant improvement showed that using the TYM-MCI provides a significant improvement in model performance compared with a model that used age, MMSE and ACE-R only.

In this study, the TYM-MCI has a sensitivity of 0.79. Therefore, used alone it will not detect some cases of aMCI/AD. Using the TYM-MCI with the ACE-R improves the sensitivity or increasing the TYM-MCI cut-off to 16/30 increases the sensitivity to 0.93.

Patients diagnosed with SMC are a heterogeneous group including patients with no organic disease, psychiatric problems or other diseases. A few patients with an original diagnosis of SMC later present with aMCI/AD: 13 patients in this study who were initially felt not to have aMCI/AD were later diagnosed with it. These 13 individuals scored significantly lower than other SMC patients on all subtests of the TYM-MCI (particularly visual recall). A low score on the TYM-MCI cautions against diagnosing SMC.

The scores on the TYM-MCI in the most difficult patients—those with equivocal ACE-R scores and patients whose diagnosis changed were similar to those of the whole aMCI/AD cohort. This suggests that the TYM-MCI score may fall early in the disease and then plateau. This corresponds with physiological and anatomic studies which show severe involvement of hippocampal and parahippocampal structures early in the disease prior to AD pathology spreading to other areas.^19^ This suggests the TYM-MCI may detect aMCI/AD prior to the development of a generalised dementia.

Another study has examined the use of the TYM-MCI in a memory clinic.^22^ The TYM-MCI was administered to 38 patients in whom the diagnosis of MCI or subjective memory impairment was uncertain. With a cut-off of 15/30, the TYM-MCI proved very sensitive for detecting MCI when the diagnosis was unclear, the author concluded that the TYM-MCI was useful in the assessment of such patients.

The current study has several limitations: the recruitment period of this study overlapped with an earlier study.^14^ The two studies had different inclusion criteria but patients with aMCI/AD could be recruited into either. There was no dual recruitment but not all patients meeting criteria were recruited into this study. Based on a comparison of results from the studies if all aMCI/AD patients had been included in this study it would have increased the number of patients in the aMCI/AD group and lowered the TYM-MCI scores of the aMCI/AD cohort further increasing the power and observed effect sizes. Nonetheless, this study was adequately powered.

The interpretation of cognitive tests depends on the clinical setting. This study was performed in a NHS clinic and patients were managed by experienced memory specialists according to their usual practice. This makes the results applicable to similar settings but there was some variation in investigative practice. The patients were diagnosed clinically with support from neuroimaging, neuropsychological assessment and follow-up in selected individuals. Use of cerebrospinal fluid (CSF) biomarkers and more specialised functional and structural imaging could support the clinical diagnosis but were not practical to use in all subjects. The diagnosis of aMCI/AD was based on international consensus criteria rather than biomarkers.

In summary, the TYM-MCI performs at least as well as the ACE-R as a cognitive test in distinguishing patients with mild aMCI/AD from those with SMC. Further studies are needed to directly compare the TYM-MCI with other short cognitive tests such as the MOCA^11^ and Qmci.^23^ The TYM-MCI is useful in the diagnosis aMCI/AD in patients with equivocal ACE-R scores and in aMCI/AD patients initially diagnosed as having SMC or unknown. Uniquely among short cognitive tests the TYM-MCI examines visual and verbal recall. The TYM-MCI shows a consistent pattern with very low scores on visual recall and low scores on verbal recall even in the mildest patients. It adds value to the ACE-R. Very few patients with aMCI/AD pass both tests and very few patients with SMC fail both tests. Importantly for clinicians the TYM-MCI is free, quick and easy to use. It takes 5 min to learn how to administer the TYM-MCI. The TYM-MCI is a very useful aid in the diagnosis of aMCI/AD, alone or in combination with the ACE-R. The TYM-MCI will be available to download free from the website tymtest.com.
