# Supplementary material for: The Test Your Memory for Mild Cognitive Impairment (TYM-MCI)
Source: J Neurol Neurosurg Psychiatry. 2017 Sep 14;88(12):1045–51. doi: 10.1136/jnnp-2016-315327 (PMC5740554; doi:10.1136/jnnp-2016-315327)
Supplement: Supplementary Appendix 1 [file jnnp-2016-315327supp001.pdf]

APPENDIX 1

Please make a copy of the drawing within the red square:

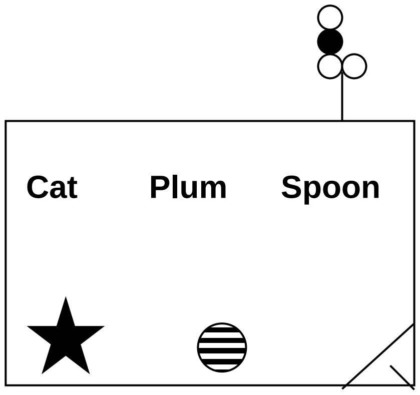

Please read the following passage carefully twice

Farmer Fred jumped onto his red tractor and drove down bluebell lane. He passed the stables with the 2 horses and nearly ran over Mrs Jones' dog. The yellow daffodils were in bloom.

He stopped by the farm gate and fed his 4 goats and admired the violets in the hedgerow. Then he walked the 200 metres to the next field and crossed the small bridge over the stream. He was pleased to see that the primroses were still in bloom. He looked across the valley to where Farmer George's 2 donkeys were grazing and then sat on the bench and ate his lunch.

Please name the 4 animals in the passage.

1.
2.
3.
4.
How many animals in total did Farmer Fred see?

Please try to remember the drawing you copied earlier and make a copy within the red square:

Please answer the following questions on the passage you read earlier:

What were the 4 animals that Farmer Fred saw?

1.
2.
3.
4.

How many animals in total did Farmer Fred see?

Please circle the flowers mentioned:

**Roses**	**Violets**	**Bluebells**	**Dandelions**
**Daffodils**	**Primroses**	**Snowdrops**	**Cowslips**

What was the name of the other Farmer?

What colour was Farmer Fred's tractor?

How far did Farmer Fred walk?

© The Queen Elizabeth Hospital King's Lynn NHS Trust, 2008. All rights reserved. Not to be reproduced in whole or in part without the permission of the copyright owner.
Website: www.tymtest.com
