# Supplementary material for: The Test Your Memory for Mild Cognitive Impairment (TYM-MCI)
Source: J Neurol Neurosurg Psychiatry. 2017 Sep 14;88(12):1045–51. doi: 10.1136/jnnp-2016-315327 (PMC5740554; doi:10.1136/jnnp-2016-315327)

APPENDIX 2

Performance of the TYM test in this cohort of Patients

The ordinary TYM test was also included in this study. In order to avoid confusion between the similarly named TYM and TYM-MCI tests the TYM results were not included in the main paper. Previous experience has shown that a patient with typical mild aMCI/AD will 'pass' the TYM test judged by overall score but has a distinctive pattern with almost full marks on all tests except recall of the sentence. This illustrates the importance of examining the pattern of scoring on a short cognitive test as well as the overall score.

The TYM test was administered as described in [12].

TABLE A1

Characteristics and results of the 6 study groups

Group	Diagnosis	No. of patients	Mean age	MMSE /30	ACE-R /100	TYM /50	TYM-MCI /30
1	SMC	96	59.2	28.8	90.8	45.9 (sd3.2)	19.8
2	Mild AD	58	67.8	26.8	81.3	40.6 (sd4.5)	9.4
3	No clear diagnosis	13	66.5	27.1	82.0	41.9 (sd5.0)	14.9
4	Fixed deficits	11	61.7	27.6	84.0	42.0 (sd6.6)	13.4
5	Other neurological disease	12	61.9	28.5	88.2	44.2 (sd4.1)	16.8
6	Non-AD dementia	10	66.6	27.4	82.8	39.4 (sd6.3)	14.0

TABLE A2
Comparing the two groups on the Mann-Whitney U test

	Mean scores of patients with SMC	Mean scores of patients with amnestic MCI/AD	Percentage change	P value Mann Whitney U test
MMSE	28.6 (sd 1.1)	26.8 (sd 1.5)	7	<0.001
ACE-R	90.8 (sd 5.5)	81.3 (sd 5.3)	10	<0.001
TYM TEST	45.9 (sd 3.2)	40.6 (sd 4.5)	11	<0.001
TYM-MCI	19.8 (sd 5.0)	9.4 (sd 4.6)	53	<0.001

For the TYM test the AUC for the ROC was 0.82 with a sensitivity of 0.60 and specificity of 0.80 using the usual cut-off of 42. The PPV was 0.71 and NPV 0.75. Using the age-matched groups the results for the TYM test remain highly significant (p<0.001 Mann Whitney U test). In this group the sensitivity was 62% and specificity was 84%.

Analyzing the pattern of scoring on the TYM test, the overall score showed a significant difference between patients with aMCI/AD and patients with no neurological cause for their cognitive complaints. But the absolute difference between the two groups is relatively small and over half of the difference is accounted for by the differences in the scores on recall of the sentence.

Using the TYM in combination with the TYM-MCI showed that very few patients with SMC failed both tests. Seven out of nine SMC patients who failed the TYM-MCI passed the TYM. However, most patients with aMCI/AD (3/12) who passed the TYM-MCI also passed the TYM. So with the combination of the TYM-MCI and the TYM there were very few false positives, but addition of the TYM to the TYM-MCI did not alter the false negative rate significantly.

CONCLUSION

The TYM test, a multi-domain test, does not detect all patients with aMCI/AD on the overall score, but analysis of the pattern of scoring detects the large majority of cases. Used in combination with the TYM-MCI it reduces the false positive rate much more than the false negative rate. This suggests that the best combination of tests for detecting the mildest AD is the ACE-R with the TYM-MCI.

Appendix Figure Box plots to show distribution of TYM

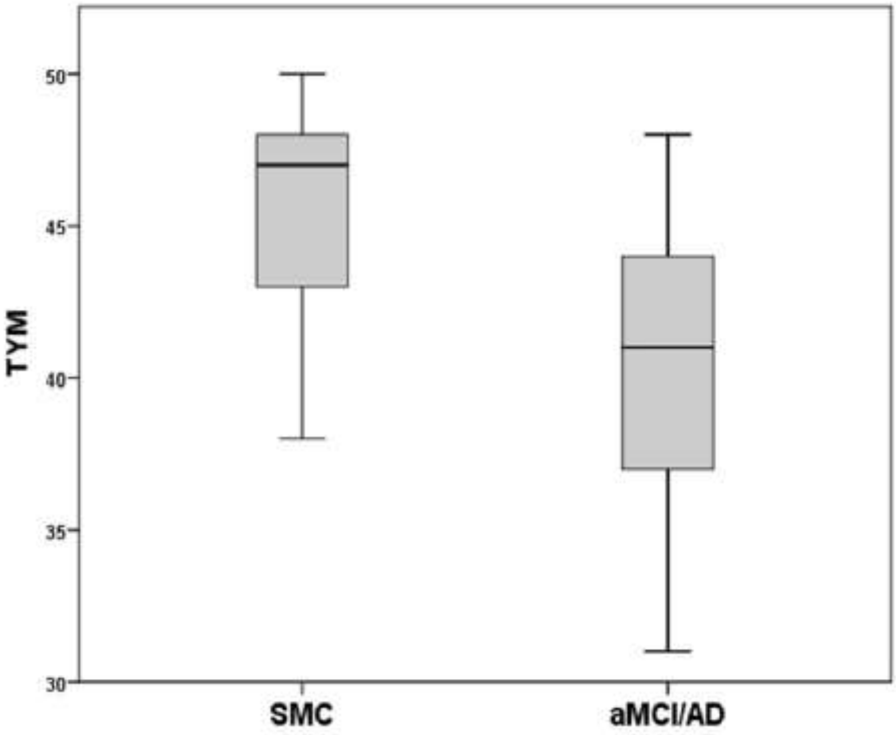

Supplement: Supplementary Appendix 2 [file jnnp-2016-315327supp002.pdf]
